# The Use of Soundscapes in Environmental Education: Teachers’ Competencies in Auditory Analysis and Emotional Identification

**DOI:** 10.3390/bs15060744

**Published:** 2025-05-28

**Authors:** José Salvador Blasco-Magraner, Pablo Marín-Liébana, Amparo Hurtado-Soler, Ana María Botella-Nicolás

**Affiliations:** 1 Department of Didactics of Physical, Artistic and Music Education, University of Valencia, 46010 Valencia, Spain; pablo.marin-liebana@uv.es (P.M.-L.); ana.maria.botella@uv.es (A.M.B.-N.); 2 Department of Experimental and Social Sciences Education, University of Valencia, 46010 Valencia, Spain; amparo.hurtado@uv.es

**Keywords:** sustainability, soundscape, environmental education, teacher training, acoustic perception, emotional responses, music education

## Abstract

Sustainability has gained significant importance in today’s educational context due to growing environmental concerns. This highlights the need to address this concept in teacher education so that future educators are equipped to help students develop competencies in this area. This study explores the use of soundscapes in environmental education, focusing on exploratory listening in relation to natural and urban sounds within the context of sustainability. The study examined the ability of 151 pre-service teachers (62 in music education and 89 in general education) to identify, characterize, and respond emotionally to these sounds. The methodology included an ad hoc task based on Schafer’s principles to identify sound elements, the PANAS questionnaire to assess emotional states before the listening activity, and a questionnaire to evaluate the emotions experienced during the task. The results indicate limited competence in characterizing sound elements, with music education students performing better than their general education peers. Furthermore, natural environments were associated with positive emotions, while urban environments elicited negative feelings, with no significant differences between the two groups. These findings underscore the importance of integrating soundscape awareness into university curricula to promote greater environmental consciousness and emotional well-being.

## 1. Introduction

Social changes related to sustainability are having a significant impact on education, driven by growing concerns about the environment and the future of the planet. This shift has influenced the way teaching, learning, and thinking are approached in educational institutions (Aggarwal, 2023; Eskici, 2023). According to UNESCO (2014), education on climate change enhances environmental awareness and concern, fostering behavioral change by encouraging civic engagement. This requires adapting educational programs and environments to meet sustainability needs, ensuring that educational systems address topics such as climate change, natural resource conservation, human rights, and social justice (Sidiropoulos, 2014).

In this context, students must not only be taught content related to sustainability but also be encouraged to adopt sustainable practices within educational settings, such as efficient resource use, recycling, and responsible consumption in schools (Biasutti, 2015). University faculty, likewise, must continue developing their sustainability competencies to effectively address current environmental and social challenges (Busquets et al., 2021; Lozano-Díaz et al., 2023; Varga et al., 2007). Sustainability competencies encompass not only environmental protection but also social and economic dimensions that are essential to a holistic education (Noguera-Méndez et al., 2024). Sustainability competencies are understood as a set of skills, knowledge, and attitudes that enable individuals to address environmental, social, and economic challenges responsibly and consciously. These competencies include critical thinking about the impact of our actions, decision-making that promotes long-term sustainability, and active participation in initiatives that foster equity and environmental conservation (Brundiers et al., 2020; Wiek et al., 2011).

Teachers are encouraged to integrate these concepts into their courses, stay updated on scientific and pedagogical advances, and develop cross-cutting competencies such as critical thinking and problem-solving (Membrillo-Hernández et al., 2021; Sammalisto et al., 2015). In addition, teachers can serve as role models for social responsibility and institutional commitment, while also promoting sustainability through academic research (Ralph & Stubbs, 2013).

Several studies have examined sustainability competencies among prospective primary education teachers (Guo et al., 2024), highlighting the generally low levels of environmental competence in pre-service teacher populations (Álvarez-García et al., 2018; Espinosa & Ortega, 2022). Other research has attempted to address this gap by incorporating soundscape and visual landscape approaches into teacher training (Hurtado et al., 2023). Moreover, some studies suggest that students themselves are calling for a deeper transformation of higher education to foster sustainability-oriented competencies (Lambrechts et al., 2013).

Since sustainability affects all areas of life, it must be broadly integrated into the university curriculum and embedded across diverse educational contexts (Dmochowski et al., 2016; Hill & Wang, 2018; Taşdemir & Gazo, 2020). Achieving this goal requires strengthening institutional processes that ensure effective implementation and fulfillment of sustainability commitments (Brito et al., 2018). In line with this cross-disciplinary approach, the present article focuses on the importance of auditory and emotional perception in educational contexts, specifically examining how pre-service teachers interact with the sounds in their environment. This interaction goes beyond the mere aesthetic appreciation of sound, also addressing a fundamental aspect of sustainability: the ability to analyze the sonic elements of the environment, which allows for the identification of noise pollution and promotes a soundscape that supports the cognitive and emotional development of individuals (Thompson et al., 2022). In this context, exploratory listening emerges as an essential skill for recognizing, raising awareness of, and modifying soundscapes within the framework of sustainability. This concept presents a way of listening that involves a high cognitive load, fostering focused attention and greater perceptual openness, rather than relying on pre-established knowledge frameworks (Reybrouck et al., 2024). In this sense, exploratory listening is an active and open way of interacting with sound, characterized by curiosity and the willingness to discover new auditory elements. This approach views music not merely as a collection of pre-existing pieces, but as a “sound environment” that invites attention and discovery. This attitude is particularly relevant in the experience of the soundscape, where auditory stimuli do not necessarily follow a conventional musical structure but emerge from the complexity and diversity of the environment. The authors integrate theories from pragmatic philosophy, such as those of Dewey (1980) and James (1890), to emphasize the importance of direct experience and the construction of knowledge through interaction with sound. For all these reasons, working on exploratory listening in various educational settings becomes an essential goal for training more conscious and critical individuals regarding their sonic environment.

In this regard, the connection between environmental listening and sustainability lies in the ability to perceive, interpret, and respond to the sonic dimension of our surroundings in a conscious and reflective manner. Soundscapes reflect the ecological and social conditions of a given environment; excessive noise, for instance, often signals unsustainable urban development, while the presence of natural sounds can indicate ecological balance. By fostering environmental listening, teachers can help students develop greater sensitivity to these auditory cues, leading to increased awareness of issues such as noise pollution, biodiversity loss, and acoustic equity. Listening, therefore, becomes a tool not only for aesthetic or emotional engagement, but also for critical reflection and action in favor of more sustainable and just environments. This approach is particularly relevant in the context of sustainability education as it promotes direct sensory engagement with the environment, encouraging learners to make connections between sound, place, and ecological responsibility.

Accordingly, this article pursues three main objectives: (1) to analyze pre-service teachers’ ability to identify and analyze sound elements within different soundscapes; (2) to examine whether there are differences in auditory perception between generalist teachers and music education specialists; and (3) to explore the emotional responses associated with the various sound elements present in these soundscapes.

### 1.1. Soundscape in Environmental Education

The concept of the soundscape, developed by Canadian composer and musician R. Murray Schafer, goes beyond understanding sounds as mere perceptual elements. This approach also recognizes them as pedagogical tools and factors that influence students’ overall well-being within learning environments (Schafer, 1993; Truax, 2021). Integrating soundscapes into education opens the door to exploring how environmental sounds impact students’ attention, concentration, creativity, and emotional well-being (Akita, 2023; Han et al., 2023; Lubman & Sutherland, 2002; Shu & Ma, 2019). For this reason, the study of soundscapes is also present in environmental education and sustainability programs, aiming to raise awareness about the significance of sound and its influence on how we perceive our surroundings. Many universities have therefore incorporated studies on the impact of soundscapes into their campus planning and sustainability efforts (B. Barrett et al., 2024; Liang et al., 2025; Mancini et al., 2021).

While the Canadian school of thought has played a foundational role in soundscape studies, it is also important to acknowledge the contributions of American composer Pauline Oliveros, particularly through her concept of “Deep Listening”. This practice, which combines focused attention, meditation, and improvisation, promotes a heightened awareness of the sonic environment and its emotional impact. Oliveros’ work broadens the pedagogical potential of soundscapes by emphasizing the act of listening as a transformative and reflective process, fostering not only ecological awareness but also emotional intelligence and empathetic engagement with the environment (Oliveros, 2005).

From an educational perspective, soundscapes can be understood through the lens of experiential learning, particularly in relation to the model proposed by Kolb (1984), which emphasizes the importance of direct experience as a starting point for reflection and knowledge construction. Activities such as soundwalks, acoustic mapping, or sound diaries offer students opportunities to actively engage with their sonic environment, facilitating concrete exploration that later transforms into reflection and conceptual understanding. This learning approach, based on sensory participation and lived experience, is especially relevant in the field of environmental education, as it fosters an integrated ecological awareness that involves not only the cognitive dimension but also emotional and perceptual aspects.

On the other hand, the integration of soundscapes can also be examined through Bronfenbrenner’s ecological systems theory (Bronfenbrenner, 1979), which posits that human development takes place in constant interaction with various levels of the environment. The soundscape belongs to the microsystem, as it represents an immediate and sensory dimension of the student’s environment, but it is also influenced by broader contexts such as the exosystem (e.g., urban planning policies or institutional decisions) and the macrosystem (cultural and social values associated with noise or silence). This systemic view allows us to understand soundscape competence not only as an individual skill, but as a capacity deeply embedded in the ecological context in which it is developed, highlighting how what we hear both shapes and is shaped by our relationship with the environment.

In this article, listening competencies are approached through the concept of soundscape competence, defined as the ability to interpret, recognize, and critically engage with the auditory environment. This competence involves much more than identifying individual sounds; it requires interpreting their meaning, recognizing acoustic patterns and structures, and understanding how sound shapes our perception of space. In essence, it calls for a critical and reflective approach to reading and listening to the surrounding soundscape (Truax, 1996). From this perspective, soundscape competence plays a key role in fostering environmental awareness, acoustic sensitivity, and the development of auditory perception skills (Bruce & Davies, 2014; Gao et al., 2023). It also highlights the importance of addressing noise pollution, drawing attention to its effects on human health, concentration, and overall well-being, and emphasizing the need to reduce intrusive noise (Taborda et al., 2020). Accordingly, sustainability programs that incorporate soundscapes seek to promote healthier urban environments by prioritizing natural sounds and mitigating noise pollution (Hong et al., 2020; Renterghem et al., 2020). At the same time, participatory approaches to soundscape education encourage students to actively analyze their sonic environments, helping them understand how sound influences perception and emotional well-being (Botella & Ramos, 2024).

Furthermore, incorporating soundscape elements into efforts to naturalize university environments reinforces the idea that educational spaces should be more than just functional; they should promote well-being and environmental consciousness (Lamanauskas, 2023; Malik, 2024; Yang et al., 2022; Zhou, 2024). The use of soundscapes on university campuses can contribute significantly to this aim, while also producing restorative effects (Hurtado & Botella, 2023; Liang et al., 2025).

To better interpret students’ emotional responses to soundscapes, it is useful to integrate psychological theories of emotion. The cognitive appraisal theory (Lazarus, 1991; Scherer, 2001) explains that emotions arise from how individuals assess the personal relevance of a stimulus. In this view, a soundscape’s emotional impact depends not only on its acoustic qualities but also on the listener’s interpretation. Lisa Feldman Barrett’s theory of constructed emotion (L. F. Barrett, 2017) adds that emotions are not fixed states but are shaped by past experiences, bodily sensations, and cultural context, highlighting the subjective nature of soundscape perception. Complementing this, the circumplex model of affect (Russell, 1980) classifies emotions along two axes: valence (pleasant–unpleasant) and activation (high–low), offering a nuanced framework to map emotional reactions to sounds (e.g., calm, alert, anxious). Finally, attention restoration theory (Kaplan & Kaplan, 1989) supports the idea that natural soundscapes foster emotional recovery and well-being by gently capturing attention and reducing cognitive fatigue.

### 1.2. Soundscape and Sustainability in Music Education

In music education, the soundscape plays a key role in building sustainability competencies. By integrating soundscapes into music education, students not only develop musical skills but also become more aware of sustainability issues and their natural surroundings (Foster & Sutela, 2024; Shevock & Bates, 2019).

Soundscapes help students become more attuned to the environment by allowing them to hear and understand the sounds of nature. This auditory awareness fosters emotional connections with ecosystems and highlights the human impact on the environment (Beatrix et al., 2024; Dumyahn & Pijanowski, 2011; Hurtado et al., 2022, 2023). In addition, soundscape education promotes the appreciation of sonic heritage, teaching students the value of preserving environmental sounds as part of our cultural and natural heritage (Ng & Tan, 2024; Wang, 2023). It also stimulates creativity and critical thinking by encouraging students to use natural sounds in composition and to reflect on environmental issues such as climate change and biodiversity loss (Botella & Ramos, 2024; Bylica, 2020). Likewise, soundscape work supports sustainability through musical practice, encouraging the use of sustainable instruments and the performance of music in natural settings (Arbuthnott & Sutter, 2019; Song et al., 2022).

Moreover, music education students develop a deeper understanding of the significance of sound and its connection to emotions, creativity, and environmental perception (Bidelman et al., 2011, 2014; Micheyl et al., 2006; Song et al., 2024; Strait et al., 2010). This underscores the importance of using soundscapes as a tool to foster sustainability competence among pre-service teachers.

## 2. Materials and Methods

### 2.1. Participants

This study involved 151 pre-service teachers enrolled in the fourth year of a Bachelor’s Degree in Elementary Education at a public university in Spain, including 62 specializing in music education and 89 in general education. Participants ranged in age from 20 to 26 years, and 84.21% identified as female. A non-probabilistic sampling method was used to select four class groups during the 2023–2024 and 2024–2025 academic years. Participation was voluntary, and no compensation was provided. All participants were informed of the study’s objectives and gave their consent to take part. Confidentiality of responses and anonymity in data processing were ensured throughout. The study was conducted in accordance with fundamental ethical principles in scientific research, following current regulations and respecting the autonomy and dignity of all participants.

### 2.2. Procedure

Initially, the participants were welcomed into a computer lab, where each had access to a personal computer. The objectives of the research were explained to them, and they signed an informed consent form. Subsequently, they completed the PANAS questionnaire online to measure their initial emotional state. Following this, the participants were given a field notebook in which they had to freely select 4 scenes from a total of 35, belonging to 4 different types of soundscapes, as shown in Table 1. The soundscapes used were those most commonly found in the city where the study took place, a large and bustling coastal city bordered by nearby mountains and characterized by a historic agricultural area that forms a green belt around the municipality. Thus, each participant analyzed one scene from each of these soundscapes, which are representative of their local cultural environment.

Each scene included a link to a video that the participants were required to watch. Each of the 35 videos had been previously recorded by the research team in a 360° format using Samsung VR (Seoul, Republic of Korea) and GoPro Fusion cameras (San Mateo, CA, USA) and edited with Gear 360 Action Director (Seoul, Republic of Korea), GoPro Fusion Studio 1.3, and GoPro VR Player 3.0. These videos were available for open access on YouTube and represented characteristic landscapes of the Eastern Mediterranean, featuring a variety of sound elements. After the immersive listening and viewing of each scene, the participants completed an exploratory listening task where they had to identify each sound element, describe its characteristics, and fill out a questionnaire where they noted the emotions experienced during each listening.

### 2.3. Data Collection Instruments

To examine the participants’ ability to identify and characterize soundscapes, an ad hoc task was designed based on the principles proposed by Schafer (1993). The task included one open-ended question in which the participants were asked to identify the sonic elements present in each soundscape, along with four multiple-choice or dichotomous items. These items required the participants to classify each sound based on its source (natural, human, or technological), frequency (continuous, repetitive, or singular), intensity (loud or soft), and perceived location (distant, close, or mid-range). To facilitate this process, the participants were provided with a table and instructed to complete it so that each row corresponded to one of the identified sounds (Table 2).

The PANAS questionnaire (Watson et al., 1988), as translated by Sandín et al. (1999), was used to assess emotional states prior to the listening activity. This instrument consists of 20 items divided into two categories according to emotional valence: 10 items measure positive emotions and 10 measure negative emotions. Responses are rated on a five-point Likert scale ranging from “very slightly or not at all” (1) to “extremely” (5). The PANAS is designed to measure the intensity of emotions or feelings experienced by participants at the time of assessment, aiming to capture their immediate emotional states. This questionnaire was selected as the primary tool for data collection due to its low cost and proven effectiveness in gathering this type of information across large sample sizes. Additionally, previous studies, such as those by Fincham and Linfield (1997) and Watson et al. (2000), support the internal consistency and structural validity of the scale.

To explore the emotions experienced during the soundscape listening activity, an open-ended question was included, prompting the participants to describe the emotions elicited by each of the sonic elements.

### 2.4. Data Analysis

In the analysis of teaching competencies, the normality of the variables was assessed using the Kolmogorov–Smirnov test, which yielded non-normal results. Consequently, the nonparametric Mann–Whitney U test was used to examine differences between generalist students and those specializing in music education. Rosenthal’s rank biserial correlation coefficient was employed as a measure of effect size and interpreted following Cohen’s (1988) guidelines: 0.1–0.3 (small), 0.3–0.5 (moderate), and >0.5 (large). Results were standardized on a 10-point scale.

Initial emotional equivalence was analyzed using the Student’s t-test. To examine the emotions experienced during the listening activity, the participants’ open-ended responses were standardized and, whenever possible, adapted to align with the PANAS emotion categories. Descriptive data were extracted in the form of relative frequencies. Differences between groups were assessed using the chi-square test of association. Effect sizes were calculated using Cramer’s V and interpreted according to the thresholds proposed by Cohen (1988).

## 3. Results

The results present the observed listening exploratory competencies of the pre-service teachers. They are divided into two sections: soundscape analysis and the experienced emotions during the virtual immersion.

### 3.1. Teaching Competencies in Soundscape Analysis

It was found that the pre-service teachers exhibit a generally low competence in analyzing soundscapes (M = 5.70, SD = 1.26). Specifically, their ability to identify elements and discriminate their production was higher than in the other variables (Table 3). Descriptively, it can be observed that the participants studying music education scored better than the general education students across all variables although they still showed medium to low scores.

As shown in Table 4, significant overall differences were found between the generalist and music education students in the variables of identification (*p* = 0.012), production (*p* = 0.021), frequency (*p* < 0.001), intensity (*p* < 0.001), and localization (*p* = 0.009), as well as in the total score (*p* < 0.001). In all cases, the music education students scored higher. Effect sizes were moderate for frequency (r = 0.442), intensity (r = 0.337), and total score (r = 0.406) and low for identification (r = 0.240), production (r = 0.220), and localization (r = 0.252).

Despite the overall differences across all the variables analyzed, these were not observed in all the types of soundscapes used in the study. In the case of the coastal soundscape (Table 5), significant differences were found in all variables: identification (*p* = 0.001), production (*p* = 0.001), frequency (*p* < 0.001), intensity (*p* = 0.038), localization (*p* = 0.035), and total score (*p* < 0.001). The effect sizes were moderate for identification (r = 0.343), production (r = 0.343), frequency (r = 0.478), and total score (r = 0.417) and small for intensity (r = 0.232) and localization (r = 0.231). For the mountain soundscape (Table 6), only marginal differences were found in localization (*p* = 0.066), with a small effect size (r = 0.195).

Regarding the orchard soundscape (Table 7), significant differences were observed in frequency (*p* = 0.026), with a small effect size (r = 0.248), and marginally significant differences were found in the total score (*p* = 0.071), also with a small effect size (r = 0.203). For the urban soundscape (Table 8), significant differences were found in frequency (*p* = 0.040), intensity (*p* < 0.001), localization (*p* = 0.023), and total score (*p* = 0.003), with marginally significant differences in identification (*p* = 0.053) and production (*p* = 0.051). The effect sizes were moderate for intensity (r = 0.369) and total score (r = 0.336) and small for identification (r = 0.211), production (r = 0.213), frequency (r = 0.225), and localization (r = 0.250). In all of the aforementioned cases, the students specializing in music education scored higher than the general education students.

### 3.2. Emotional Experimentation

The PANAS test only revealed significant differences between groups in the emotions of distressed, t(157) = 2.23, *p* = 0.027, and inspired, t(157) = −2.81, *p* = 0.006, while no differences were found in the other 18 emotions (Table 9). Therefore, it can be stated that the emotional equivalence of the groups was maintained prior to completing the questionnaire related to emotional experimentation.

**Figure 1:**
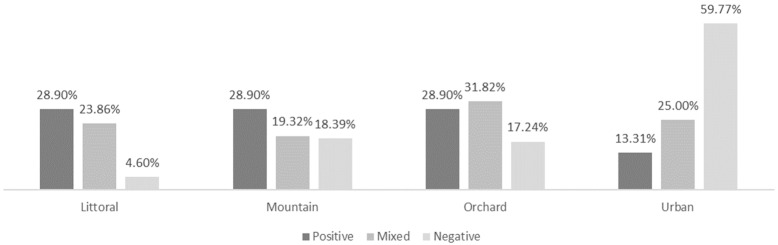
Experienced emotions.

As expected, the emotions experienced by the pre-service teachers were more positive in the coastal, mountain, and orchard landscapes and more negative in the urban landscape (Figure 1). Specifically, in the coastal landscape, they reported feelings of tranquility/calm/relaxation when listening to the sea (67.74%) and the breeze (33.26%), irritation from human voices (12.90%), and pleasantness from birds (11.83%). In the mountain landscape, they indicated tranquility/calm/relaxation with the sound of the wind (33.95%) and water (14.68%) and irritation from the sound of insects (13.76%) and engines (19.27%). In the orchard landscape, they experienced tranquility/calm/relaxation from birds (32.77%) and the wind and leaves (16.81%), a pleasant sensation from birds (22.69%), and irritation from engines (26.89%) and voices (9.24%). In the urban landscape, they reported experiencing irritation/agitation from the noise of people (46.79%) and engines (33.11%) and tranquility from the sound of birds (11.01%). When analyzing whether there were significant differences in emotional experiences between the music and generalist groups (Table 10), differences were only observed in the orchard landscape (χ^2^ = 9.03, *p* = 0.011), with a small effect size (V = 0.275).

## 4. Discussion

This study examined the exploratory listening competencies of pre-service teachers in various soundscapes, with the aim of investigating potential differences in auditory perception between generalist teachers and those specialized in music education. Additionally, the emotional impact that these soundscapes had on the participants was evaluated. First, the results reveal that the pre-service teachers show limited competence in analyzing soundscapes. This finding suggests the need for training in this area, as suggested by Wang (2023), who implemented a project with university students to develop their skills in musical interpretation and creation, thereby improving their perception of soundscapes. Similarly, Botella and Ramos (2024) conducted an intervention with secondary school students, highlighting the need to strengthen environmental education through an ecological, modern, pragmatic, and humanistic approach. In this context, the integration of experiential methodologies is particularly relevant. According to Kolb’s experiential learning model (Kolb, 1984), meaningful learning arises from the interaction between concrete experience, reflective observation, abstract conceptualization, and active experimentation. Activities such as soundwalks, acoustic mapping, or listening diaries would allow pre-service teachers to engage directly with their acoustic environment, reflect on their auditory experiences, and construct more robust understandings of soundscapes. This kind of embodied and reflective learning process supports the development of soundscape competence and encourages a more active role in sustainability education.

This type of learning, which emphasizes direct experience with the environment, can be linked to Akita’s (2023) study, which found notably high results in soundscape perception among students in Tokyo. This finding suggests that constant exposure to the urban sound environment, particularly in a city with high levels of noise pollution, may have heightened the students’ sensitivity to their sonic surroundings. In this sense, the students’ experience of noise could be viewed as an informal, yet impactful, form of experiential learning, which, as proposed by Kolb (1984), could lead to deeper engagement with and understanding of the soundscapes around them. However, it is important to note that despite this possible sensitization, Song et al. (2022) found no correlation between the level of acoustic noise and students’ assessments of soundscape perception. This indicates that while environmental factors may play a role, the development of soundscape competence likely involves more complex dynamics than exposure to noise alone.

In this regard, Bruce and Davies (2014) argue that expectations of a soundscape are influenced by previous experiences, which condition our perception of space. This aligns with Truax’s (1996) concept of soundscape competence, which suggests that individuals are more adept at recognizing sounds from familiar soundscapes, such as urban environments, in contrast to less common environments like orchards. This phenomenon can be better understood through Bronfenbrenner’s ecological systems theory (Bronfenbrenner, 1979), which explains human development as the result of interaction with various environmental levels. Within this framework, soundscapes are part of the student’s microsystem, their immediate sensory environment, but they are also influenced by broader factors such as urban policies (exosystem) or cultural values related to sound (macrosystem). Thus, soundscape competence is understood as a skill constructed within a complex ecological context.

From a psychological perspective, the way individuals emotionally respond to soundscapes is shaped by more than the acoustic environment itself. The cognitive appraisal theory (Lazarus, 1991; Scherer, 2001) suggests that listeners evaluate the meaning of what they hear, determining whether it is relevant to their goals, safety, or comfort. A natural soundscape may be interpreted as calming by one person but as melancholic by another, depending on prior experiences. Similarly, Barrett’s theory of constructed emotion (L. F. Barrett, 2017) also states that emotions do not arise automatically, but are formed based on what we feel in our bodies, the environment we are in, and what we have learned culturally. These theories highlight the subjective nature of emotional responses and help explain why the participants in this study reported varied emotional reactions to the same soundscapes.

The circumplex model of affect (Russell, 1980) is also useful for analyzing the emotional data collected as it allows the classification of feelings according to levels of arousal and valence. For example, natural landscapes in this study were typically associated with low arousal and high valence emotions (e.g., calm, contentment), while urban soundscapes generated high arousal and low valence emotions (e.g., irritation, anxiety). This framework enables a more refined interpretation of the PANAS results and the qualitative emotional responses of the participants.

Furthermore, attention restoration theory (Kaplan & Kaplan, 1989) provides a cognitive explanation for the emotional benefits of natural soundscapes. It suggests that listening to soft sounds, such as flowing water or rustling leaves, helps restore attention and reduce mental fatigue, which also improves mood and emotional well-being. These restorative qualities were clearly reflected in the participants’ more positive emotional assessments of natural environments compared to urban ones.

In any case, following the line of Hurtado et al. (2023), it is essential to promote competence in soundscape analysis among future teachers. This would provide a more holistic education on sustainability, enabling them to foster greater sensitivity to and understanding of the sonic environment in their own students (Guo et al., 2024). This is also consistent with the research of Álvarez-García et al. (2018), who assessed the environmental competencies of pre-service teachers at the beginning and end of their studies at a Spanish university, demonstrating that they lacked sufficient environmental competencies.

Second, the pre-service teachers specializing in music demonstrated greater competence in identifying and analyzing soundscape elements compared to the generalists. These results align with previous research that has shown that musicians and students of music education are more competent in identifying and analyzing soundscape elements compared to non-musicians or those without specific music training. This finding is consistent with the works of Bidelman et al. (2011, 2014), Micheyl et al. (2006), Song et al. (2024), and Strait et al. (2010), who agree that musical training has a positive impact on musicians’ perceptual sensitivity. In this regard, taking a general music course promotes richer auditory perceptions and clearer emotional associations with sound (Song et al., 2024).

However, these differences are not uniform across all types of soundscapes and are particularly marked in the variables of frequency and intensity. This is consistent with Bidelman et al.’s (2014) research, which asserts that musicians’ superior psychophysical abilities are especially evident in situations where sound is affected by background noise or interference (degraded noise). In this sense, this study also suggests that musical training leads to an improvement in tone discrimination ability, as proposed by Micheyl et al. (2006). Like Bidelman et al. (2011), we consider that enhancing musical perception benefits cognitive skills. This underscores the importance of sound in the development of auditory perception and shows that musical practice enhances skills such as attention and memory (Strait et al., 2010).

On the other hand, teacher training programs should take into account the variability of acoustic competencies based on specialization. In this regard, these programs could benefit from the inclusion of a general music course, which would promote greater auditory ability and a deeper understanding of the sonic environment (Song et al., 2024). Additionally, the implementation of practical exercises that challenge teachers to identify, categorize, and reflect on sounds in different environments could contribute to better preparation for managing the emotional and perceptual impact of soundscapes. In this regard, the inclusion of listening practices inspired by Pauline Oliveros’ concept of Deep Listening could enrich teacher training. By encouraging attentive, meditative, and emotionally engaged listening, this approach fosters not only perceptual sensitivity but also reflective and empathetic capacities in relation to the acoustic environment. These dimensions are especially relevant for future educators who must recognize the emotional nuances of the soundscapes that shape their students’ learning experiences (Oliveros, 2005).

This type of training is also essential for strengthening the emotional sensitivity of future teachers to sounds, as suggested by the studies of Liang et al. (2025) and Arbuthnott and Sutter (2019), which show that natural sounds have a positive effect on emotional well-being and cognitive performance. It would be beneficial for future teachers to analyze emotional responses to natural sounds versus urban noise, which would allow them to develop greater empathy and sensitivity to the effects of sounds on students and the community at large.

In addition to the previous recommendations, teacher training programs could integrate the concept of ecological acoustic competence into their curricula. This approach could include the study of how sounds affect behavior and social interactions, helping future teachers understand how soundscapes contribute to the psychological and emotional well-being of students (Bruce & Davies, 2014; Truax, 1996). Additionally, educational technologies could be incorporated to allow teachers to analyze and modify soundscapes, serving as tools for creating more suitable learning environments. Including activities that promote interdisciplinary collaboration, such as working with experts in music, environmental psychology, and urban planning, would also enrich the understanding of the effects of sounds in various educational and social contexts (Gao et al., 2023; Han et al., 2022).

Third, the results of this study reflected the influence of soundscapes on the participants’ emotional responses, with a clear difference between natural and urban soundscapes. At the beginning of the experiment, both groups showed emotional equivalence. However, when assessing the sensations experienced during exposure to the different soundscapes, the participants indicated more positive emotions in natural and human-made landscapes, while the emotions associated with the urban soundscape were predominantly negative. This is consistent with Liang et al.’s (2025) study, which demonstrates how natural sounds, such as wind or moving water, have a positive emotional impact on individuals, while artificial noises, such as construction or traffic, can generate adverse emotional responses.

Although the distinction between natural and urban soundscapes may seem very clear, in this study it is used as an analytical tool. This classification is based on differences that have already been observed in the structure of sound, the emotions they evoke, and how we perceive them, according to previous research (e.g., Hong et al., 2020; Han et al., 2023). Natural soundscapes tend to have softer and more constant sounds, which are associated with positive emotions and relaxation. On the other hand, urban soundscapes tend to have loud, high-pitched, and irregular noises, which can generate stress or discomfort. This difference does not mean that they are strictly opposed, but rather serves as a way to understand how different types of sounds affect people’s emotions.

The relationship between natural soundscapes and improved emotional well-being has also been documented in other studies, such as Arbuthnott and Sutter (2019), who highlight that natural environments foster emotional well-being and cognitive performance, particularly in creative reasoning tasks. Similarly, this study also found that natural soundscapes have a positive effect on the participants’ emotions.

On the other hand, the urban soundscape, dominated by artificial noises, generated higher scores in negative emotions. This phenomenon has also been observed by Hong et al. (2020), who argue that including natural sounds in urban environments, such as nature sounds, can mask unpleasant urban noises and improve the quality of the soundscape. These findings align with Han et al.’s (2023) study, which asserts that certain sound characteristics, such as roughness and fluctuation, affect our emotions. All of this suggests that natural sounds can have a positive impact as they have a smoother and more regular texture compared to urban noise.

Furthermore, Beatrix et al. (2024) propose solutions to improve urban soundscapes, such as the creation of green spaces that would not only reduce noise pollution but also improve people’s emotional well-being. This perspective is supported by Gao et al. (2023), who found that urban environments dominated by construction are typically perceived negatively in terms of acoustic quality, while natural landscapes generate more positive responses. Therefore, recommendations for designing urban soundscapes that favor emotions, such as those proposed by Han et al. (2022), are effective in mitigating the negative effects of noise in these contexts.

An interesting aspect that emerged in this study was the lack of significant differences between the two groups regarding their perception of soundscapes. This finding is consistent with Song et al.’s (2022) work, which also found no correlation between the level of acoustic noise and the students’ evaluations of soundscape perception. However, this result contrasts Song et al.’s (2024) study, which indicated that students who had taken music courses developed different emotional judgments about sound environments. This point suggests that, although musical training could influence emotional perception of soundscapes, other factors, such as individual characteristics or the context of the environment, may have a more significant impact. In this regard, previous research has shown that differences in emotional sensitivity and sound perception are not only influenced by academic training but also by factors such as personality, past experiences, and cultural expectations toward certain sounds (Bruce & Davies, 2014; Truax, 1996). Additionally, soundscapes are highly contextual phenomena, meaning that emotional responses can vary significantly depending on factors such as the urban versus natural environment, the socio-economic characteristics of the listeners, or even environmental planning policies in different areas (Han et al., 2023; Gao et al., 2023). Therefore, this variability underscores the complexity of human auditory perception, which cannot be explained solely by musical training, but also by a dynamic interaction between the individual and their sonic environment.

## 5. Conclusions

This study has used the soundscape to assess the exploratory listening competencies of pre-service teachers. The findings have shown that the auditory competence of the students varies depending on their specialization, with the music education teachers demonstrating a greater ability to identify and analyze the sound elements present in the soundscapes. However, all participants exhibited limited competence, highlighting the need to enhance these skills in both initial and ongoing teacher training programs in a transversal manner.

Additionally, it was observed that natural soundscapes generate more positive emotional responses, whereas urban environments tend to provoke negative emotions. This finding underscores the importance of integrating natural sounds into both educational spaces and urban environments to promote emotional well-being. The inclusion of experiential methodologies, such as sound walks, listening journals, or the “Deep Listening” approach, can strengthen teachers’ auditory, emotional, and ecological sensitivity, aligning with models of meaningful learning and educational sustainability.

Moreover, the results indicate that the emotional perception of soundscapes does not depend solely on musical training, but also on contextual, cultural, and personal factors, reinforcing the need for holistic education that considers the sonic dimension from an ecological perspective. The emotional responses observed in this study also reflect underlying cognitive and affective processes, which can be better understood through psychological theories that highlight the role of individual appraisal, memory, and environmental context in emotional experience. This suggests that future research should continue to explore how specific soundscape characteristics interact with these psychological dimensions to influence perception and well-being.

Among the limitations of this study, it is worth noting that the sample size was relatively small and focused on a specific context, which may limit the generalization of the results. Thus, university campuses with different urban planning layouts, located in non-urban environments, belonging to sociocultural contexts with distinct patterns of noise production and tolerance, or offering degree programs with different student needs, might yield different results. Future research should consider expanding the sample and including a broader range of educational contexts to explore how soundscapes influence emotional and academic well-being across different cultures and environments. Additionally, we emphasize the need to integrate soundscapes into sustainability education programs, especially in teacher training, to strengthen environmental competencies among students, thereby fostering greater ecological and emotional awareness.

## Figures and Tables

**Table 1:** Soundscapes used for 360° virtual immersion.

Type of Landscape	VR Collection Link	Scenery
Littoral	https://bit.ly/3rBkMtA (accessed on 20 May 2025)	Beach, beach with shells, dunes, beach with seaweed, beach sunrise
Mountain	https://bit.ly/3JOmKwN (accessed on 20 May 2025)	Daisies, life on ground (I and II), tree, herbaceous, Turche cave, pinewoods, clover, cork trees
Orchard	https://bit.ly/3KU9qsa (accessed on 20 May 2025)	Vegetable garden, school garden (I and II), garden seesaw, garden soil, among avocados, walk through fields, fruit trees, almond trees, spring garden, donkeys garden, vines, olive trees, orange trees
Urban	https://bit.ly/3EojW8t (accessed on 20 May 2025)	Traffic, medieval market, religious festival, district, fireworks, garden, amusement park

**Table 2:** Ad hoc task.

Sonic Element	Source			Frequency			Intensity		Location		
	Natural	Human	Technological	Continuous	Repetitive	Singular	Loud	Soft	Distant	Close	Mid-Range
1.											
2.											
3.											
…											

**Table 3:** Global differences between music and primary pre-service teachers. Descriptive statistics.

Group	Identification	Production	Frequency	Intensity	Location
Music	7.34 (1.49)	7.34(1.53)	5.27 (4.13)	5.79 (1.78)	4.48 (1.61)
Primary	6.81 (1.40)	6.83 (1.47)	4.13 (1.40)	4.80 (1.51)	3.73 (1.53)
Total	7.03 (1.45)	7.04 (1.51)	4.60 (1.64)	5.21 (1.69)	4.04 (1.61)

Mean (Standard deviation).

**Table 4:** Global differences between music and primary pre-service teachers. Mann–Whitney U test.

Variable	Music (Mdn/M)	Primary (Mdn/M)	U	*p*	r
Identification *	7.50/7.34	6.91/6.81	2073	0.012	0.240
Production *	7.50/7.34	6.91/4.13	2122	0.021	0.222
Frequency ***	5.36/5.27	4.14/4.13	1524	<0.001	0.442
Intensity ***	6.13/5.79	4.74/4.80	1809	<0.001	0.337
Localization **	4.17/4.48	3.75/3.73	2042	0.009	0.252
Total score ***	6.17/6.17	5.37/5.38	1621	<0.001	0.406

* *p* < 0.05, ** *p* < 0.01, *** *p* < 0.001.

**Table 5:** Differences between music and primary education pre-service teachers regarding littoral soundscape.

Variable	Music (Mdn/M)	Primary (Mdn/M)	U	*p*	r
Identification **	10.00/8.45	6.67/6.93	925	0.001	0.343
Production **	10.00/8.45	6.67/6.93	925	0.001	0.343
Frequency ***	6.67/6.89	3.33/4.63	735	<0.001	0.478
Intensity *	6.33/5.92	4.00/4.53	1082	0.038	0.232
Localization *	3.33/4.28	3.33/3.35	1083	0.035	0.231
Total score ***	7.78/6.92	5.56/5.41	821	<0.001	0.417

* *p* < 0.05, ** *p* < 0.01, *** *p* < 0.001.

**Table 6:** Differences between music and primary education pre-service teachers regarding mountain soundscape.

Variable	Music (Mdn/M)	Primary (Mdn/M)	U	*p*	r
Identification	6.67/6.48	6.67/6.36	1741	0.963	0.005
Production	6.33/6.41	6.67/6.40	1741	0.846	0.021
Frequency	4.00/4.08	3.33/3.57	1559	0.306	0.109
Intensity	5.00/5.12	5.00/4.54	1502	0.182	0.142
Localization ^†^	4.00/4.49	3.33/3.57	1408	0.066	0.195
Total score	5.14/5.40	4.86/4.96	1544	0.274	0.118

^†^ 0.05 ≤ *p* ≤ 0.10.

**Table 7:** Differences between music and primary education pre-service teachers regarding orchard soundscape.

Variable	Music (Mdn/M)	Primary (Mdn/M)	U	*p*	r
Identification	7.50/7.55	5.56/5.50	1289	0.229	0.132
Production	7.50/7.42	6.67/7.13	1317	0.302	0.114
Frequency *	5.00/4.99	3.33/3.85	1117	0.026	0.248
Intensity	6.00/5.66	4.64/4.75	1238	0.134	0.167
Localization	3.33/4.12	3.33/3.91	1410	0.651	0.051
Total score ^†^	5.83/6.09	5.56/5.50	1184	0.071	0.203

* *p* < 0.05, ^†^ 0.05 ≤ *p* ≤ 0.10.

**Table 8:** Differences between music and primary education pre-service teachers regarding urban soundscape.

Variable	Music (Mdn/M)	Primary (Mdn/M)	U	*p*	r
Identification ^†^	7.50/7.01	5.71/6.12	1205	0.053	0.211
Production ^†^	7.50/7.13	5.71/6.14	1202	0.051	0.213
Frequency *	5.00/5.63	5.00/4.30	1184	0.040	0.225
Intensity ***	7.50/6.65	5.00/5.03	964	<0.001	0.369
Localization *	5.00/5.18	3.33/3.80	1146	0.023	0.250
Total score **	6.67/6.49	5.42/5.26	1015	0.003	0.336

* *p* < 0.05, ** *p* < 0.01, *** *p* < 0.001, ^†^ 0.05 ≤ *p* ≤ 0.10.

**Table 9:** Emotional differences between groups in PANAS questionnaire.

Emotion	t	df	*p*
Interested	−1.14	157	0.256
Alert	−0.59	157	0.559
Excited	−2.09	157	0.038
Upset	−1.52	157	0.131
Strong	−0.45	157	0.657
Guilty	−1.28	157	0.201
Afraid	−0.70	157	0.485
Hostile	0.47	157	0.637
Enthusiastic	−0.99	157	0.320
Proud	−0.99	157	0.322
Irritable	1.28	157	0.200
Distressed	2.23	157	0.027
Ashamed	−0.73	157	0.468
Inspired	−2.81	157	0.006
Nervous	0.094	157	0.925
Determined	−1.28	157	0.202
Attentive	−0.70	157	0.484
Jittery	0.73	157	0.468
Active	−0.74	157	0.458
Scared	0.10	157	0.922

**Table 10:** Differences in experienced emotions.

Soundscape	Group	Positive	Mixed	Negative	Total	χ^2^	*p*	V
Littoral	Music	32	8	1	41	0.530	0.767	0.072
	Primary	44	13	33	60			
	Total	76	21	4	101			
Mountain	Music	34	6	5	45	1.29	0.525	0.109
	Primary	42	11	11	64			
	Total	76	17	16	109			
Orchard	Music	39	7	3	49	9.03	0.011	0.275
	Primary	37	21	12	70			
	Total	76	28	15	119			
Urban	Music	19	8	18	45	3.61	0.164	0.182
	Primary	16	14	34	64			
	Total	35	22	52	109			

## Data Availability

The data presented in this study are available on request from the corresponding author.
